# Performance of SD Bioline Malaria Ag Pf/Pan rapid test in the diagnosis of malaria in South-Kivu, DR Congo

**DOI:** 10.11604/pamj.2017.27.216.11430

**Published:** 2017-07-21

**Authors:** Théophile Mitima Kashosi, Joseph Minani Mutuga, Devotte Sifa Byadunia, John Kivukuto Mutendela, Basimike Mulenda, Kanigula Mubagwa

**Affiliations:** 1 Laboratory of Biomedical Research and Public Health, Faculty of Medicine & Community Health, Université Evangélique en Afrique (UEA), Bukavu, DR Congo; 2 Department of Laboratory Technologies, Institut Supérieur des Techniques Médicales (ISTM), Bukavu, DR Congo; 3 Clinical Laboratory, General Referral Hospital, Katana, DR Congo; 4 Médecins d’Afrique, Coordination-Europe, Savigny Sur Orge, France; 5 WHO, Malaria Capacity Development/Strategic Planning & Prevention, Regional Office for Africa, Libreville, Gabon; 6 International Center for Advanced Research and Training (ICART), Bukavu, DR Congo, and Department of Cardiovascular Sciences, University of Leuven, Leuven, Belgium; 7 Department of Cardiovascular Sciences, University of Leuven, Leuven, Belgium

**Keywords:** Performance, SD Bioline, Malaria Ag Pf/Pan, South-Kivu, DR Congo

## Abstract

**Introduction:**

Use of malaria rapid diagnostic tests (RDTs) has improved the management of this disease. We evaluated the validity of the SD-Bioline Malaria-Ag-Pf/Pan™ (Batch 60952) RDT supplied by the Malaria Control Program of the DRCongo.

**Methods:**

cChildren (n = 460) aged below 5 years seen in curative care (CC) for suspected malaria and in pre-school consultation (PSC) in two rural centers underwent clinical evaluation and capillary blood collection for microscopic reading of thick smear (TS) and thin film (BF), and for RDT. Sensitivity (Se), specificity (Sp), positive (PPV) and negative (NPV) predictive values of the RDT, and the corresponding accuracy and Youden indices were determined using microscopic data as reference. Results were compared using the Chi-square test.

**Results:**

Microscopy showed malaria infection in 53.8% of CC and in 10.8% of PSC children. Similar results were obtained using the RDT (CC: 47.1%; PSC: 18.3%; P > 0.05 vs. microscopy). Se of the RDT was 82.1%, Sp 92.0%, PPV 88.5% and NPV 87.4%. RDT positivity was significantly (p < 0.01) associated with some symptoms (chills, profuse sweating) and with a recent history of malaria attack. In addition, Se of the RDT depended on parasitemia and decreased at low parasite denstity.

**Conclusion:**

SD-Bioline Malaria-Ag-Pf/Pan™ RDT has a relatively good sensitivity and specificity but seems useful only for high parasitemia. Negative SD Bioline Malaria Ag Pf/Pan™ RDT should be complemented with microscopy when clinical signs suggest malaria.

## Introduction

Malaria remains a major public health concern. It is estimated that in 2013, malaria affected about 200 million people and caused 600 000 deaths [1]. Sub-Saharan Africa is particularly affected and contributes over 90% of global malarial mortality. The vast majority of victims are children under 5 years of age (78%) and pregnant women. Since 2000, increased efforts have been made to reduce the transmission of the disease and improve its treatment. Recent reports indicate that the use of mosquito bed nets impregnated with long-lasting insecticides (LLINs) and of indoor residual spraying (IRS) of insecticides as transmission prevention tools and of artemisinin-based combined drugs (ACT) for the treatment of cases has led to a significant decrease in malaria morbidity and mortality [1]. In the DRCongo, malaria is endemic over nearly the whole national territory, except in the high plateaus of the Eastern part of the country (Tanganyika district of Katanga Province, the North-Kivu and South Kivu Provinces, and the Ituri district of Orientale Province) where disease outbreaks occur in epidemic forms due to sporadic and seasonal transmission. These latter regions have also experienced population movements recently, from low to high transmission areas, and vice versa, due to wars, civil strives and persisting socio-economic crises. A pre-intervention assessment (Roll Back Malaria [RBM]/National Malaria Control Program [NMCP] Strategic Plan 2009-2013) conducted in 2007 in 71 health zones of the country showed that an average under 5 years Congolese child experienced 6-10 episodes of fever or malaria per year, that malaria was responsible for 39.3% of outpatient consultations, 39.1% of in-hospital mortality of children under 5 years. The general in-hospital mortality was estimated at 6% [2].

Early diagnosis of the disease remains the basis for improved case management. Laboratory diagnosis of malaria, classically based on microscopy, is faced with problems related to its technical implementation in resource-poor areas [3]. The current use of rapid diagnostic tests (RDTs) to detect antigens constitutes an important forward step in the diagnostic strategy because it allows parasitological diagnosis even in areas where it is impossible to have good microscopy services [4]. However, given the current lack of comparative field trials and the heterogeneity of the transmission and epidemiology of malaria in South Kivu, the reliability on results obtained by RDTs remains uncertain. The possibility of false positive results in malaria-free samples as a consequence of cross-reactions with other diseases or of other causes makes it imperative to test for the predictive values of the RDTs. In early 2010, WHO recommended that all suspected malaria cases be confirmed by RDT and/or microscopy before being treated. Diagnostic accuracy should significantly improve the quality of care and ensure a rational and appropriate use of antimalarial drugs. The existence of several RDTs on the market, the possibility of forgery, and the uncertainty about test stability during transport and storage, etc. are all reasons to reinforce quality control. WHO recommends that all test batches be checked before or after shipping by a quality control center in collaboration with the WHO and the Foundation for Innovative New Diagnostics (FIND). However, a well-functioning quality control system for RDTs is still lacking in the DRCongo, particularly in provinces such as South-Kivu. Therefore the objective of the present study was to assess the sensitivity and specificity of the SD Bioline Malaria Ag Pf/Pan™ RDT (Batch No. 60952) provided by the National Malaria Control Program (NMCP) of the DR Congo, and to compare the performance with that of a few other RDTs used in the region. The SD Bioline Malaria Ag Pf/Pan™ RDT contains a membrane strip precoated with mouse monoclonal antibodies specific to histidine-rich protein II (HRP-II) of *P. falciparum* and with mouse monoclonal antibodies specific to lactate dehydrogenase (pLDH) of four Plasmodium species (*P. falciparum, P. vivax, P. malariae and P. ovale*). The antibodies are mixed with colloid gold conjugate and react with the malaria antigen in the patients’ samples. The aim of this study is to contribute to improving the management of malaria cases by ensuring a proper diagnosis using valid and efficient tests.

## Methods

This is a cross-sectional study carried out in two Health Centers, Nuru and Ciranga, in the Katana Health Zone, from January to April 2014, in the middle of the rainy season. Our study population consisted of 460 children aged 6-59 months, including 340 seen during consultations for treatment and 120 seen during pre-school consultations, which in DRC are mandatory for health status suveillance and disease prevention (eg by vaccination) before 5 years of age [5]. We selected exhaustively all children attending the curative consultation (CC) in which a clinical examination led to suspect the presence of uncomplicated malaria. The rest of the sample was randomly selected among children attending the pre-school consultations (PSC). Only children whose mothers had agreed to participate were included in this study. Children under antimalarial treatment, those with clinical symptoms suggesting severe malaria, and those with clinical symptoms suggestive of other acute febrile diseases (e.g. measles, acute respiratory infections, ear infections, tonsillitis, abscesses, acute diarrheal disease) or of underlying chronic diseases (e.g. tuberculosis, HIV/AIDS) were excluded from the study. Demographic, clinical and laboratory data were recorded on a data collection sheet. Each case included in the study underwent capillary blood sample collection on which the following tests were carried out: RDT; thick smear (TS) and thin blood film (BF) to identify the presence of malaria parasite and its species, and to estimate the parasite density. RDT (test kits from MT Promedt Consulting GmbH. Ingbert, Germany) was performed according to instructions from the manufacturer included with the test batch. TS and BF were done on one same slide for each individual. The TS and BF samples were stained with buffered 10% Giemsa, and were read on the microscope. RDT and a first reading of TS and BF were carried out at the Laboratory of the Katana General Referral Hospital. Further slide readings for species identification and estimation of parasite density were performed in the Laboratory of Biomedical Research and Public Health (Faculty of Medicine, Université Evangélique en Afrique) and in the Laboratory of the Institut Supérieur des Techniques Médicales in Bukavu. To ensure internal quality control of the microscopic diagnosis, each TS and BF sample was read independently (without knowing the results of the other readings) at the three examination sites. The laboratory staff (including the first author of this article) examining the slides had undergone specific trainings (organized by Malaria Care Project and/or by NMCP) for microscopic diagnosis of malaria. In case of discrepancy between the results of the three independent microscopic readings, the slide was reexamined in common and the results discussed and decided between the three persons responsible for microscopy. We used MedCalc sofware for calculating sensitivity (Se), specififity (Sp), positive predictive (PPV) negative predictive values (NPV) and confidence interval at 95% (95% IC) for each value. Accuracy was calculated as: (TP+TN)/(TP+TN+FP+FN). The Youden index (designed to take into account both Se and Sp in assessing the validity of a test) was given by: Se+Sp -1.

Data were analyzed using Epi Info and XL STAT 2014 (Addinsoft, New York, NY, USA). The chi-square test was used for comparison of results. The threshold for statistical significance was set at p < 0.05. Parasite density was calculated using the following formula [6, 7]: (the formula is not displayed)

## Results

Table 1 presents the demographic and physical characteristics of the children. The whole children population average age was 31.5 ± 16.4 months, and the average weight was 12.1 ± 3.8 kg. Children in the PSC group were older than those of the CC group. Fever was present in practically all CC children, and in 10% of PSC children. On microscopic examination malaria infection was present in 53.8% of children presenting for CC and in 10.8% of PSC children. Using RDT, malaria was diagnozed in 47.1% of the CC children and in 18.3% of PSC children (Table 1; P > 0.05 for RDT vs microcopy). *Plasmodium falciparum* was the most frequently found parasite species, and the only species found in PSC children (Table 2). In no case was P. vivax identified in our population. Using microscopic results as standard, the SD Bioline RDT had a sensitivity (Se) of 82.1% (95%-CI: 76.1-86.9%) and specificity (Sp) of 92.0% (95%-IC: 88.1-94.8%). PVV and NPV were 88.5 and 87.4, reepectively. The accuracy was 0.88 and the Youden index 0.74. The RDT false positive rate was 11.5% and the false negative rate 12.6% (Table 3), with a Cohen’s index (Kappa factor, K; [8]) of the concordance between the two tests of 0.75. SD Bioline RDT proved to be less sensitive (Se: 40%; 95%-CI: 32.5 - 48.0) for the differential diagnosis of *P. falciparum* in our study (Table 4). In contrast, it is specific to 93.5% (95%-CI: 90.2 - 95.8%), with an accuracy rate of 0.76 and a low Youden index (0.34). This test shows good validity in the diagnosis of two other malaria species, namely *P. malariae* and *P. ovale*, with Se of 91.3 (95%-CI: 79.1 - 97.1) and Sp of 85.5% (95%-CI: 81.8 - 88.6) (accuracy 0.86 and Youden index of 0.76) for each species. We noticed a large statistically significant dependence (P < 0.0001) of the positivity of the RDT on the parasite density. Se of the RDT was 100% for parasite densities above 200/μm2 but decreased at lower parastiemias (Table 5). A multivariate analysis (Table 6) indicates that positive RDT is correlated with the presence of sweating (p < 0.00001), recent history of malaria (p < 0.00001), presence of chills (p = 0.0002), and presence of physical asthenia (p = 0.022).

**Table 1:** Socio-demographic and anthropometric characteristics

Characteristics	CC (n=340)	PSC (n=120)	Total (%)
**Age (31.5 ± 16.4 months)**			
< 12 months	62 (18.2)	4 (3.3)	66 (14.3)
13 - 24 months	108 (31.8)	24 (20.0)	132 (28.7)
> 24 months	170 (50.0)	92 (76.7)	262 (57.0)
**Sex ratio** (male/female)	1.04	0.81	0.97
**Weight (12.1 ± 3.8 Kg)**			
5 - 10 kg	183 (53.8)	19 (15.8)	202 (44.0)
11 - 15 kg	123 (36.2)	38 (31.7)	161 (35.0)
16 - 20 kg	32 (9.4)	56 (46.7)	88 (19.0)
> 20 kg	2 (0.6)	7 (5.8)	9 (2.0)
**Temperature (37.8 ± 1.05°C)**			
≤ 37 °C	3 (0.9)	108 (90.0)	111 (24.1)
> 37°C	337 (99.1)	12 (10.0)	349 (75.9)

CC=curative consultation. PSC=pre-school consultation. Data show that practically all children presenting for curative care had axillar temperature higher than 37 °C.

**Table 2:** Microscopy results

	Children		
	CC (n=340)	PSC (n=120)	
Microscopy result	Number (%)	Number (%)	Total (%)
Positive TS/BF	183 (53.8)	13 (10.8)	196(42.6)
Negative TS/BF	157 (46.2)	107 (89.2)	264(57.4)
**RDT results**			
Positive RDT	160 (47.1)	22 (18.3)	182 (39.6)
Negative RDT	180 (52.9)	98 (81.7)	278 (60.4)
**Plasmodium species**			
*Plasmodium falciparum*	137 (74.9)	13 (100)	150 (76.5)
*Plasmodium malariae*	36 (19.7)	-	36(18.4)
*Plasmodium ovale*	10 (5.4)	-	10(5.1)

BF: blood film. CC: curative care. PSC: pre-school consultation. TS: thick blood smear.

The data indicate more frequent positive thick smear in CC than PSC and show *Plasmodium falciparum* as the species most frequently found.

**Chi-square = 5.82, p = 0.12 for RDT vs microscopy.**

**Table 3:** RDT validity to diagnose malaria

RDT	Standard method (TS)			Se(%)95%IC	Sp(%)95%IC	PPV(%)95%IC	NPV(%)95%IC	Accuracy	Youd
	Posit.	Neg.	Tot.						
Positive	161	21	**182**	82.14 76.0-87.2	92.05 88.1-95.0	88.46 82.9-92.7	87.41 82.9-91.0	0.87	0.74
Negative	35	243	**278**						
**Total**	**196**	**264**	**460**						

Number of positive and negative results on microscopy (TS, thick blood smear) and rapid diagnostic test (RDT) are shown. Accuracy and Youden index were calculated (see formulae in Methods) from the row and percentage values.

**Table 4:** RDT Validity to diagnose *P. falciparum* malaria

RDT	*P. falciparum*			Se (%)95%IC	Sp (%)95%IC	PPV (%)95%IC	NPV(%)95%IC	Accuracy	Youd.
	Posit.	Neg.	Tot.						
Positive	60	20	**80**	40.00	93.55	75.00	76.32	0.76	0.34
Negative	90	290	**380**	32.1-48.3	90.2-96.0	64.0-84.0	71.7-80.5		
**Total**	**150**	**310**	**460**						

Number of positive and negative results on microscopy (for detection of *P. falciparum*) and rapid diagnostic test (RDT) are shown. Accuracy and Youden index were calculated (see formulaie in Methods) from the row and percentage values. Notice the low Se for detecting *P. falciparum*.

**Table 5:** Dependence of RDT result on parasite density

RDT	1-199	≥ 200	Total	Chi^2^	DF	P-value
Positive	1	160	161	189.4	2	<0.0001
Negative	35	0	35			
**TOTAL**	**36**	**160**	**196**			

Chi^2^: chi (χ) square. DF: degree of freedom

**Table 6:** Association of clinical signs/symptoms and RDT results

	RDT		Univariate analysis			Multivariate analysis		
Signs	Positive	Negative	OR	95%-CI	P value	OR	95%-CI	P value
**Recent malaria**								
Yes	31	16	3.36	1.78 -6.34	0.00009	17.43	7.00-43.38	<0.00001
No	151	262						
**Abundant sweating**								
Yes	138	45	16.23	10.19-25.85	<0.00001	6.67	3.49-12.75	<0.00001
No	44	233						
**Stiffness**								
Yes	159	87	15.17	9.15-25.15	<0.00001	2.22	0.78-6.30	0.13
No	23	191						
**Asthenia**								
Yes	160	94	14.23	8.54-23.71	<0.00001	3.13	1.17-8.32	0.022
No	22	184						
**Shivering**								
Yes	102	25	12.90	7.79-21.36	<0.00001	3.20	1.71-5.98	0.0002
No	80	253						

OR: odd ratio. 95%-CI: 95% confidence interval.

## Discussion

In the present study we examined the ability of the SD Bioline RDT to diagnose malaria in children. The population of our study consisted of two groups of children from a rural environment where malaria occurs by epidemic outbreaks and malnutrition is endemic [9]. Children consulting for curative purposes were the majority in our study. The lower inclusion of children using pre-school consultation services is explained by the fact that in Katana, despite the compulsory character of the preschool consultation children are brought to these services less frequently after their first birthday, when they have already completed the vaccination program. In addition, some mothers were reluctant to allow capillary blood collection from their children considered healthy. Microscopy identified *P. falciparum* as the most frequent malaria agent, followed by *P. malariae*, with no case of P. vivax. These results are different from those obtained in rural areas of Senegal [10] among 478 children aged 1 to 14 years, where the species distribution for *P. falciparum, vivax, ovale, malariae* was 82.8%, 7.4%, 6.0% and 2.1%, respectively. This might be explained by a difference in parasite carriage by anopheles, or by a difference in genetic characteristics (e.g. in the Duffy gene) in the populations studied. It has been noted that P. vivax is absent in Duffy gene-negative populations such as those inhabiting most of Central Africa (however see [11]) but present in Duffy gene positive populations living in Northern Ethiopia and in parts of Sudan [12]. Our results show a prevalence of malaria infection in > 10% of asymptomatic children. This high prevalence of asymptomatic carriers in our study population is larger than the one (1.0%) reported in a different area in Katana [9], but similar to the prevalence (9.5%) observed in pregnant women (E. Bahizire, unpublished). The reason for this discreapancy was not clarified but could in part be related to the seasonal epidemic occurrence of the disease outbreaks. Nevertheless this indicates that malaria remains a public health problem in Katana, despite the efforts being carried out by the Ministry of Health through its national malaria control program, such as the distribution of LLINs and the provision of effective antimalarial drugs such as ACTs. These results corroborate those of a survey conducted in Kimbaseke, Kinshasa, which found a *P. falciparum* asymptomatic carrier rate of 21% among pregnant women [13].

In the present study, SD Bioline RDT sensitivity (82.1%) and specificity (92.0%) did not reach the values indicated by the manufacturer (99.7%). The false positive rate (11.5%) is high compared with WHO standards that require a false positive rate of less than 10% [4, 14, 15]. This could be explained by the difference between populations used to estimate these parameters. In the present study, we evaluated a population with all levels of parasitemia, whereas the manufacturer´s tests were done on samples with a density of > 200 parasites/μl. Our results also differ from those found in Mont Ngafula, Kinshasa, which included 281 children aged 6-59 months with fever: Se was 99.4%, Sp 67.5%, positive PPV 78.9% and NPV 98.8% [16], but this is in an area where malaria is endemic. The SD Bioline RDT provides results that are closer to those obtained using TS, when compared to other RDTs, e.g. the Parachek RDT, which in a study conducted in Mbuji Mayi, DRCongo, found poor agreement (K = 0.41). Kappa in our study is closer to that found in Burkina Faso (K = 0.63) using Combo RDT [17]. Differences between the various studies might be explained by differences that may exist between the study populations, parasite antigenic variability between populations and environments, products batches but also by the effect of the RDT distribution and conservation chain. Our results agree with those of the Kinshasa study mentioned above [16] as far as the asymptomatic population is concerned (Se, Sp, PPV and NPV of 94.3%, 79.6%, 62.1% and 97.5%, respectively). In contrast to the manufacturer claim of 99.7% sensitivity, our results indicate a significantly poorer validity (Se: 40%) of the SD Bioline RDT to detect *P. falciparum* in our population.The performance for *P. falciparum* is relatively lower than that found by WHO in 2012 for suspected malaria in Madagascar (Se and Sp of 92.9% and 98.9%) [18]. Similar results were obtained in a study conducted in Kinshasa on children under 5 years (Se, Sp, PPV and NPV for TDR SD Bioline 93.6%, 81.1%, 60.6% and 97.6% [19]). Part of the reason of the lower general performance of the test in our study is likey related to the inclusion of low parasite densities, including the positive cases in PSC children, in which *P. falciparum* was the only parasite species. The the SD Bioline RDT is highly sensitive to parasite densities above 200 parasites par μl of blood but loses specificity to distinguish between species, as high parasite density is frequently interpreted as poly parasitism. Because the SD Bioline RDT considers many cases of high parasite density as indicating poly-parasitism and because any polyparasitism case is considered as severe malaria, there is a clear risk to disorient the prescription of ACT. In both health centers (Nuru and Ciranga) several patients were hospitalized and treated with quinine perfusion based on the diagnosis made using the SD Bioline TDR. This carries the risk of promoting the emergence of strains resistant to quinine due to bad indications. Our results are also consistent with those found in the Philippines in 2002, in which a recent history of malaria history correlated with a positive RDT or a detectable parasite density [20].

## Conclusion

This study shows that the use of SD Bioline Malaria Ag Pf/Pan ™ RDT to detect malaria parasites is more effective when the parasite density is high. This RDT loses its validity for low parasitemia. These results confirm that RDTs are valid in the diagnosis of malaria if they are complemented by microscopy. The SD Bioline remains a screening test only and is useful for large population screening, with diagnosis in individual cases suspected on infection based only on clinical evaluation requiring confirmation by microscopy. It is recommended that the NMCP should ensure the assessment and monitoring of the various RDTs offered by the different support organizations before adopting them in the health system.

### What is known about this topic

- Rapid diagnostic tests (RDTs) are widely used for point-of-care diagnosis of Malaria in resource-limited settings;
- Malaria rapid diagnostic tests (RDTs) has improved the management of this disease;
- The existence of several RDTs on the market, the possibility of forgery, and the uncertainty about test stability during transport and storage, etc. are all reasons to reinforce quality control.

### What this study adds

- Our Study shows the performance of Malaria RDTs in Eastern Democratic Republic of the Congo (DRC);
- Negative Malaria RDT should be complemented with microscopy when clinical signs suggest malaria;
- Positive RDT is correlated with the presence of sweating, recent history of malaria, and presence of chills.

## Competing interests

The authors declare no competing interest.
